# L-2-Hydroxyglutaric Aciduria Due to the Homozygous Variant c.905C>T in L2HGDH Without Cognitive Deficits or Gait Disturbance: A Case Report

**DOI:** 10.7759/cureus.107757

**Published:** 2026-04-26

**Authors:** Josef Finsterer, Awini Barwari

**Affiliations:** 1 Neurology, Neurology and Neurophysiology Center, Vienna, AUT

**Keywords:** l-2-hydroxyglutaric aciduria, leukoencephalopathy, metabolic defect, myopathy, seizures

## Abstract

L-2-hydroxyglutaric aciduria (L2HGA) is a rare, slowly progressive, autosomal recessive neurometabolic disorder caused by mutations in the L2HGDH gene and characterized by psychomotor developmental delay, cognitive impairment, epilepsy, dystonia, cerebellar ataxia, tremor, dysarthria, pyramidal signs, macrocephaly, leukoencephalopathy, and elevated L-2-hydroxyglutaric acid levels. To date, no case has been reported of a patient with L2HGA caused by the c.905C>T variant in L2HGDH presenting with epilepsy, tetraspasticity, tetraataxia, dysarthria, and dysmorphism, but without cognitive impairment or gait disturbance.

The patient is a 22-year-old man from Syria with L2HGA, born to consanguineous parents, who presented with rare generalized seizures, mild tetraspasticity, tetraataxia, dysarthria, facial dysmorphism, and unilateral cryptorchism. His psychomotor development was normal. Cerebral MRI at the age of 18 revealed marked leukoencephalopathy, but no atrophy or involvement of the basal ganglia. Surprisingly, he showed no cognitive impairment, gait disturbances, or macrocephaly. He led an independent life as a mechanic and drove his own car.

This case broadens the phenotypic spectrum of L2HGA and demonstrates that the c.905C>T variant in the L2HGDH gene can manifest phenotypically with only mild symptoms and without cognitive deficits or gait abnormalities. It also shows that some of these features may not be present in childhood but may develop only during adolescence or adulthood.

## Introduction

L-2-hydroxyglutaraziduria (L2HGA) (OMIM #236792) is a rare, slowly progressive, autosomal recessive neurometabolic disorder caused by mutations in the L2HGDH gene on chromosome 14q22.1 [1,2]. L2HGDH encodes mitochondrial 2-hydroxyglutarate dehydrogenase, a flavin adenine dinucleotide (FAD)-dependent enzyme that oxidizes L-2-hydroxyglutarate to alpha-ketoglutarate in various mammalian tissues [3]. The prevalence of L2HGA is estimated at 1 in 1,000,000 [1]. Phenotypically, the disorder is characterized by early (within the first year of life) or late-onset psychomotor developmental delay, intellectual disability, epilepsy, dystonia, cerebellar ataxia, tremor, dysarthria, pyramidal signs, macrocephaly, subcortical leukoencephalopathy, and an elevated concentration of L-2-hydroxyglutaric acid in all body fluids, particularly in urine [1,2]. Cerebral MRI shows T2 hyperintensities predominantly in the anterior subcortical white matter and the basal ganglia, with the subcortical U-fibers classically being affected [4]. The characteristic centripetal spread of lesions in the white matter, sparing the brainstem and corpus callosum, is the hallmark of cerebral imaging [4]. The diagnosis is typically made by detecting elevated L-2-hydroxyglutaric acid via urine gas chromatography-mass spectrometry and identifying a genetic defect in the L2HGDH gene [1]. There is no curative treatment for L2HGA, which is why therapy is primarily supportive and includes specific measures such as flavin adenine dinucleotide (FAD) sodium, L-carnitine chloride, antispasmodics, and antiseizure medications (ASMs) [5]. Since the first description of L2HGA in 1980, >100 patients with mutations in the L2HGDH gene have been reported [6]. Different ages of onset and severity levels of L2HGDH have been described, ranging from a neonatal onset with severe neurological deficits to a moderate-to-severe form beginning in childhood, to a mild form in adulthood [1]. To our knowledge, a patient with L2HGA due to the homozygous c.905C>T variant in the L2HGDH gene, presenting with epilepsy, tetraplegia, tetraataxia, dysarthria, and dysmorphism but without cognitive impairment, has not been described to date.

## Case presentation

The patient is a 22-year-old man who was diagnosed with L2HGA at the age of four following generalized status epilepticus and the detection of elevated levels of L-2-hydroxyglutaric acid in his urine, as well as the newly described homozygous variant c.905C>T (p.Pro302Leu) in the L2HGDH gene.

**Figure 1:**
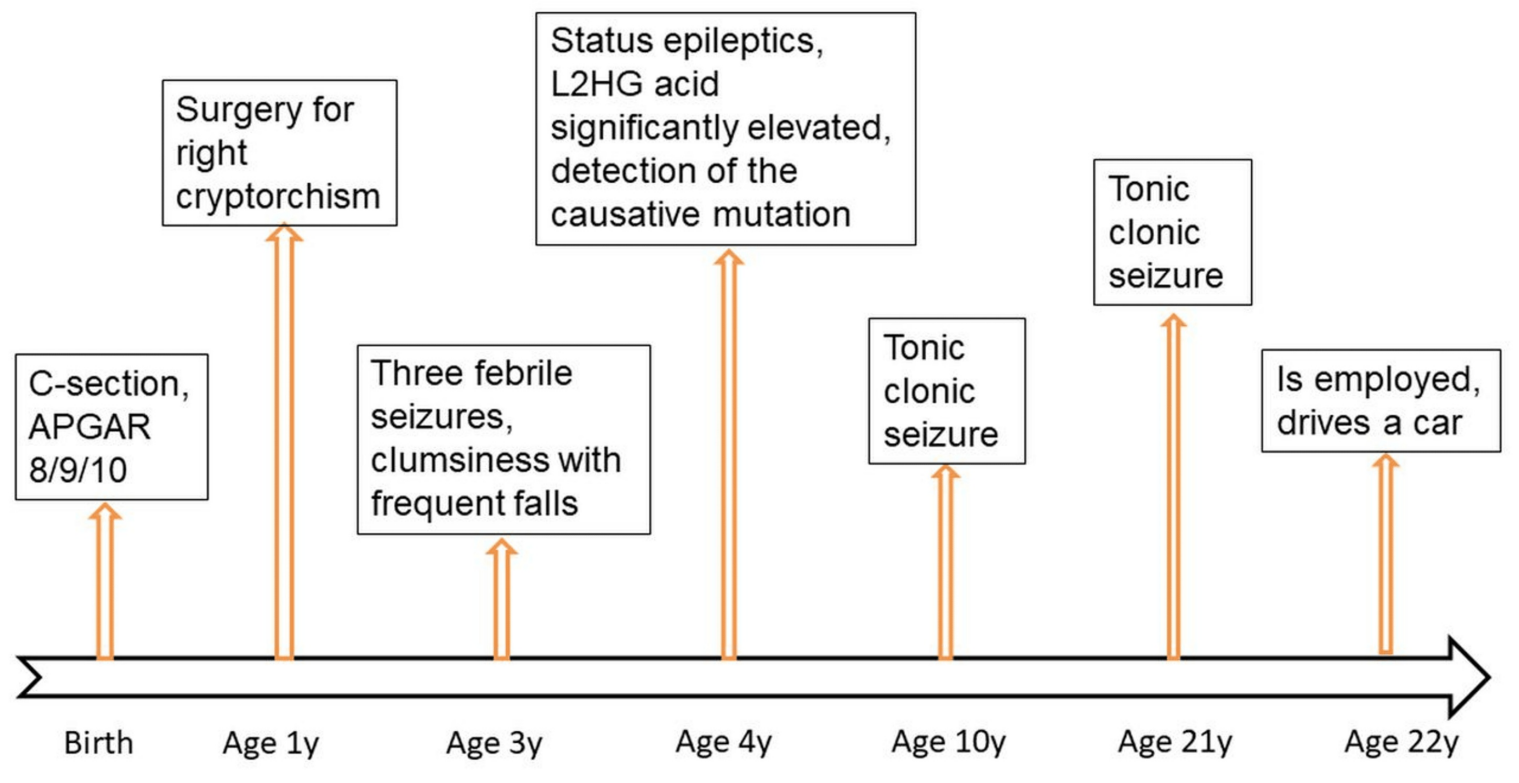
Chronological progression of the clinical presentation of the index patient (x-axis: years, y-axis: clinical manifestations)

The pregnancy proceeded without complications, but the patient was delivered by C-section due to prolonged labor and abnormal cardiotocography. The APGAR score at birth was 8/9/10. No postnatal complications occurred. He learned to sit at 7 months, to walk with support at 12 months, and to walk independently at 13 months (Figure 1). He began speaking multi-word sentences at 20 months of age. The medical history also noted an undescended right testicle (cryptorchism), which required orchidopexy at one year of age, as well as three febrile seizures - one at three years of age and two at four years of age (Figure 1). Since the age of three, motor clumsiness associated with occasional falls had been noted (Figure 1).

At the age of four, he experienced a generalized status epilepticus. The clinical neurological examination at that time was unremarkable and revealed only a questionable, intermittent squint. L-2-hydroxyglutaric acid levels were markedly elevated, whereas D-2-hydroxyglutaric acid levels were normal. The medical history revealed no evidence of developmental delay, stagnation, or regression. Cognitive development was normal. The MRI showed extensive, confluent areas of T2 hyperintensity with predominant involvement of the subcortical frontal and bilateral temporal lobes. L-carnitine was prescribed, but no ASM.

The patient experienced his next tonic-clonic seizure at the age of 10 and another at the age of 21. Repeated electroencephalograms (EEGs) were unremarkable on each occasion. The electrocardiogram and ophthalmological examination also revealed no abnormal findings.

**Figure 2:**
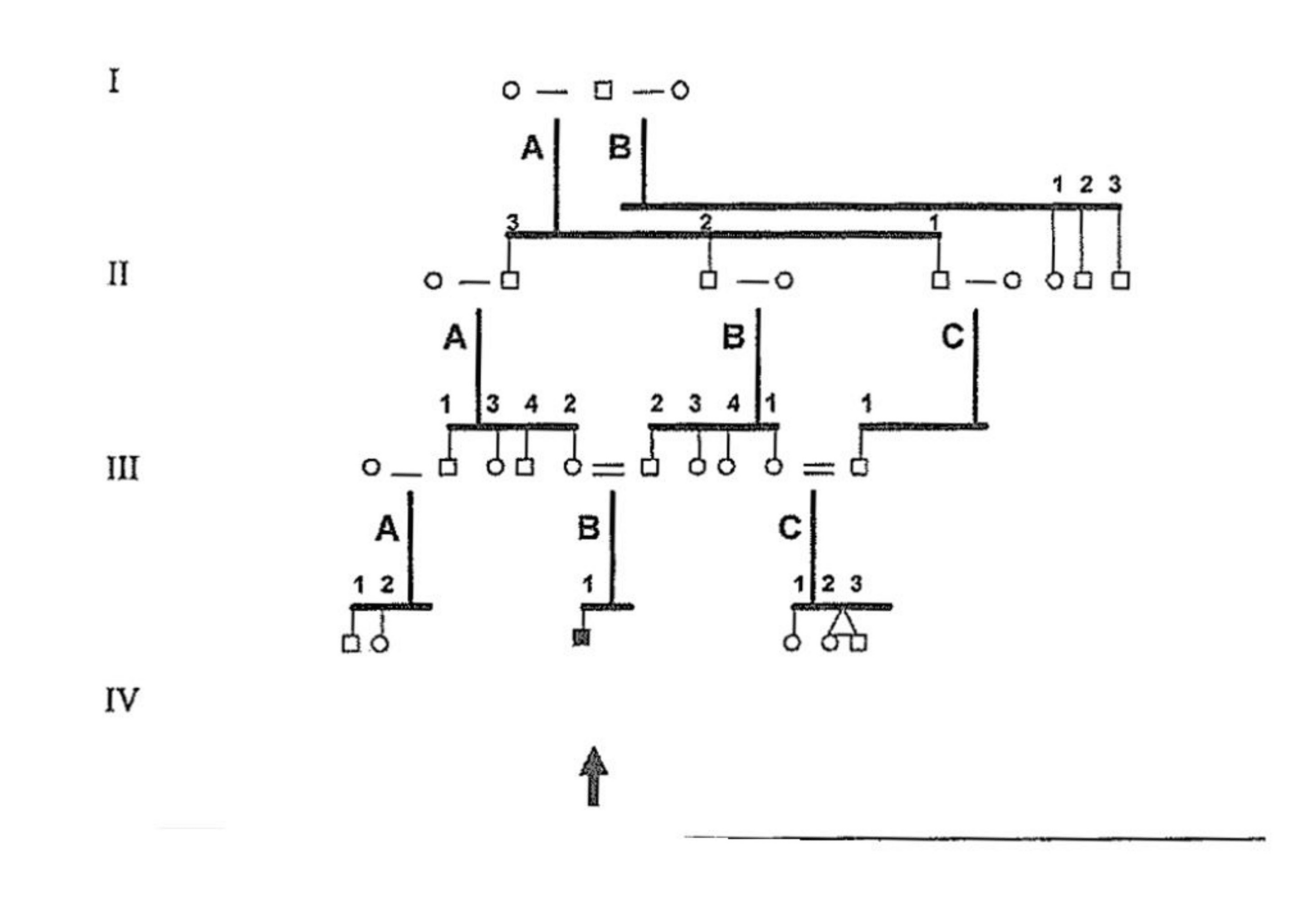
Pedigree of the index patient’s family

The family history revealed no evidence of L2HGA in any other family member; however, the index patient’s parents were blood relatives (Figure 2). One of the patient’s sisters exhibited a fine motor skills disorder. Both parents carried the c.905C>T variant in a heterozygous form but were clinically and biochemically unremarkable.

The clinical-neurological examination at age 22 revealed widely spaced eyes, dysarthria, a deep voice, an exaggerated masseter reflex, mild diffuse muscle atrophy in all four limbs, mild tetraspasticity, exaggerated deep tendon reflexes with bilateral clonus of the Achilles tendon reflexes, mild tetraataxia, and positive pyramidal signs (Table 1). The Romberg and Unterberger tests were unremarkable, and he was able to walk without assistance and without difficulty or risk of falling. There was no microcephaly or macrocephaly, and there was no apparent cognitive impairment. The Mini-Mental State Examination (MMSE) score was 29 [7].

**Table 1:** Phenotypic presentation of previously described patients with an H2LGDH mutation and the index patient

Phenotype	Previously reported	Index patient
Psychomotor delay	yes	no
Cognitive impairment	yes	no
Tremor	yes	no
Dystonia	yes	no
Cerebral neoplasm	yes	no
Microcephaly	yes	no
Gait disturbance	yes	no
Epilepsy	yes	yes
Ataxia	yes	yes
Dysarthria	yes	yes
Pyramidal signs	yes	yes
Leucoencephalopathy	yes	yes
Tetraspasticity	no	yes
Widely spaced eyes	no	yes
Cryptorchism	no	yes

**Figure 3:**
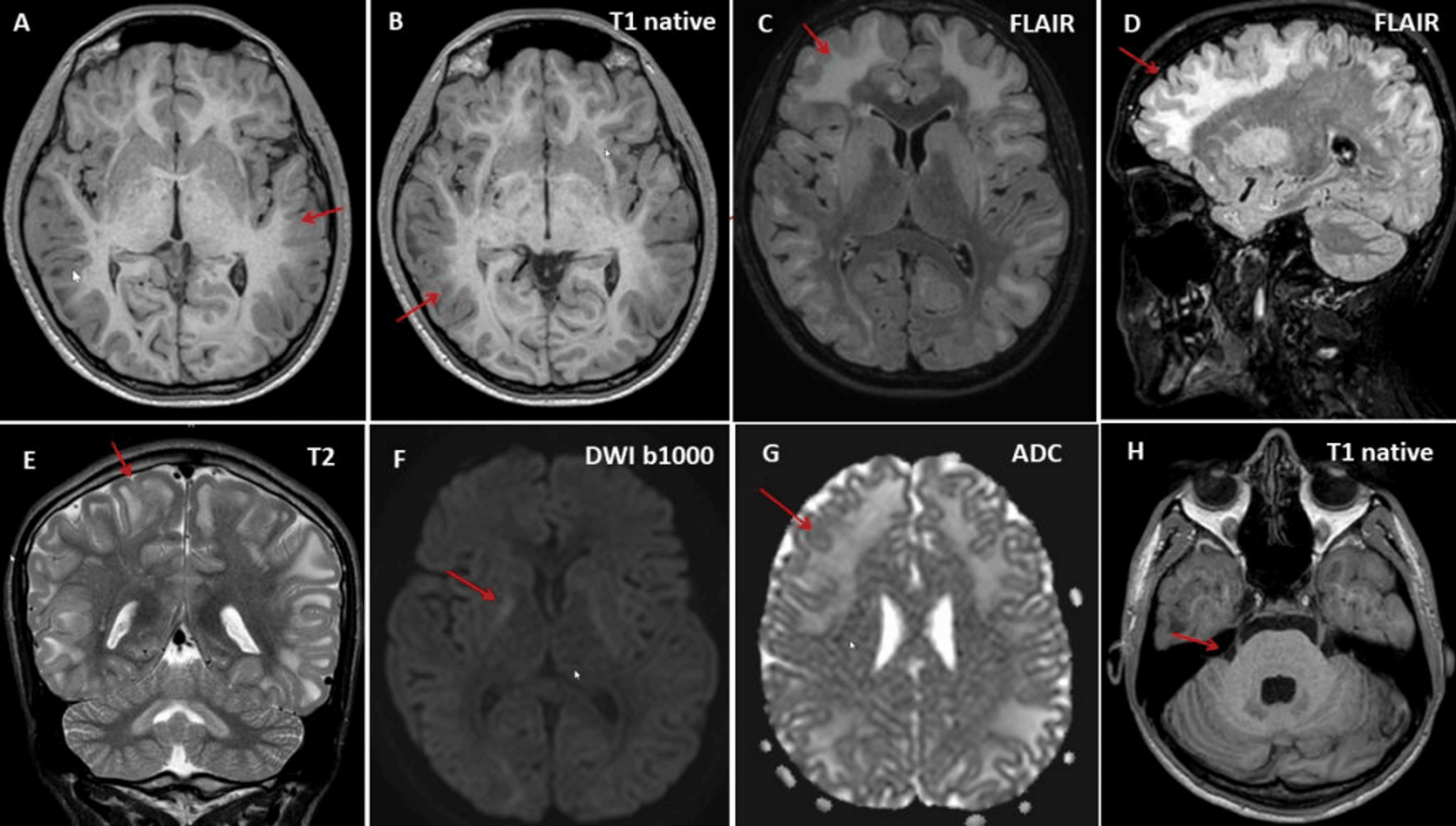
An MRI scan of the brain performed at the age of 18 showed extensive, partially confluent hyperintensities in the subcortical white matter bilaterally on T1, T2, FLAIR, and DWI (panels A-E, G), with a predominance in the frontal lobes and less pronounced in the brainstem (Panel H). These were not visible in the DWI scan (Panel F). FLAIR: fluid-attenuated inversion recovery; DWI: diffusion-weighted imaging

Blood tests revealed a mild vitamin D deficiency but were otherwise unremarkable. Brain MRI showed generalized, extensive, subcortical white matter lesions concentrated in the frontal region, with no contrast enhancement or hyperintensity on diffusion-weighted imaging and no atrophy (Figure 3). Motor-evoked potentials, recorded from the abductor digiti minimi and the abductor hallucis, showed normal motor latencies. The only medication he was currently taking was levetiracetam at a dose of 500 mg/day, adjusted for his body weight. He was currently working as an auto mechanic and held a driver’s license.

## Discussion

The patient with L2HGA presented here is of interest for several reasons. First, he is one of the few patients with L2HGA who carried the c.905C>T mutation in the L2HGDH gene. The first two patients with L2HGA who carried the c.905C>T variant were described in 2004 from Turkey [8]. The most important clinical manifestations in these patients were intellectual disability, cerebellar ataxia, gait disturbances, intention tremor, short attention span, and hyperactivity [8]. Additional findings included macrocephaly as well as febrile or afebrile, and partial or generalized seizures [8]. The third patient with L2HGA carrying the c.905G>T mutation was from Portugal and was described in 2005 [9]. He was compound heterozygous and additionally carried the c.169G>A variant [10]. Unfortunately, the individual phenotype was not described in detail [9]. The fourth L2HGA patient, who carried the c.905C>T variant, was described in 2008 from Germany [10]. He carried the mutation in a homozygous form, but the individual phenotype was not specified in detail for this patient either [10]. In a study of 10 L2HGA patients from Turkey, 5 carried the c.905C>T variant [11]. All exhibited cognitive deficits [11]. From this entire group of L2HGA cases, it was reported that most individuals showed normal development during the first year of life and later developed psychomotor retardation or regression, as well as the classic L2HGA phenotype.

Another interesting aspect of the index case is that the patient exhibited phenotypic features that have not yet been reported. These include widely spaced eyes, tetraspasticity, and cryptorchism (Table 1). In contrast, previously described L2HGA patients manifested with psychomotor retardation, cognitive impairment, epilepsy, dystonia, ataxia, tremor, dysarthria, pyramidal signs, gait disturbances, leukoencephalopathy, macrocephaly, and increased excretion of L-2-hydroxyglutaric acid (Table 1). Factors that could contribute to the variable phenotypic expression are unknown, but it can be surmised that this is due to an interaction between the mutated gene, the rest of the genome, epigenetic modifications, and environmental factors--a phenomenon known as variable expressivity or incomplete penetrance [12].

Third, the patient showed no cognitive impairment. Unlike the other patients with the c.905C>T variant [8-11], the index patient showed no developmental delays, arrests, or regression, and has not developed intellectual impairment, at least to date. Since most of the previously reported patients with L2HGA exhibited cognitive deficits [13,14], it would have been expected that the index patient would also show intellectual impairment. In a study of 10 L2HGA patients, 80% exhibited intellectual disability [11]. In contrast to these patients, the index patient completed primary and secondary school in his country without difficulty, completed an apprenticeship as an automotive mechanic, and earned a vocational certificate. He also drove a car, though under the condition that he undergo annual neurological examinations due to his epilepsy and to assess the likelihood of seizures. Why the patient was able to lead an independent life despite pronounced leukoencephalopathy is unknown, but most likely the same factors that contributed to phenotypic heterogeneity were also responsible for the absence of cognitive deficits.

The fourth point is that the seizure frequency was low. During the first 22 years of his life, he had only 6 seizures and required only the lowest effective dose of an antiepileptic drug (500 mg of levetiracetam per day, adjusted for his reduced body weight). Typically, seizure frequency is higher in patients with L2HGA [15]. The reason why seizure frequency was low despite the marked increase in L-2-hydroxyglutaric acid and the pronounced cerebral white matter lesions is unknown; however, it can be surmised that this is due to predominantly subcortical involvement of the white matter and possibly to the semi-benign phenotype of the respective L2HGDH variant.

Limitations of the study included the fact that only a few family members were genetically tested, that the conclusions were based on a single patient, and that the patient did not undergo a comprehensive neuropsychological examination.

## Conclusions

In summary, this case suggests that the homozygous c.905C>T variant in the L2HGDH gene can manifest phenotypically as mild tetraspasticity, mild tetraataxia, wide-spaced eyes, dysarthria, cryptorchism, and epilepsy, but without developmental delay, arrest, or regression, cognitive impairment, or gait disturbance. It also demonstrates that some of these features may not be present in childhood but may develop only during adolescence or adulthood. Clinicians should be aware that L2HGA can present with only mild abnormalities and allow these patients to lead a nearly normal life.
